# Demagnetization Effect in a Meander-Core Orthogonal Fluxgate Sensor

**DOI:** 10.3390/mi12080937

**Published:** 2021-08-09

**Authors:** Shaotao Zhi, Xuecheng Sun, Qiaozhen Zhang, Jie Chen, Xiangfen Zhang, Hongyu Chen, Chong Lei

**Affiliations:** 1 College of Information, Mechanical and Electrical Engineering, Shanghai Normal University, Shanghai 200234, China; zhist@shnu.edu.cn (S.Z.); zhangqz@shnu.edu.cn (Q.Z.); jiechen@shnu.edu.cn (J.C.); xiangfen@shnu.edu.cn (X.Z.); 2 Research and Development Center of Microelectronics, School of Mechatronic Engineering and Automation, Shanghai University, Shanghai 200444, China; mark_white@shu.edu.cn; 3 Key Laboratory of Thin Film and Microfabrication Technology (Ministry of Education), Department of Micro/Nano Electronics, School of Electronic Information and Electrical Engineering, Shanghai Jiao Tong University, Shanghai 200240, China

**Keywords:** demagnetization effect, orthogonal fluxgate, meander-core, finite element modeling

## Abstract

Demagnetization effect plays an important role in the magnetic core design of the orthogonal fluxgate sensor. In this paper, a meander-core orthogonal fluxgate sensor based on amorphous ribbon is described. The demagnetization model of meander-core structures is established, and the average demagnetization factor can be evaluated by finite element modeling. Simulation and experimental analyses were performed to study the effects of demagnetization on the sensitivity and linear range of orthogonal fluxgate sensors in the fundamental mode by varying the number of strips, the line width, and the spacing of the meander-cores. The results were compared and revealed a very close match. The results show that the demagnetization factor increases with an increase in the number of strips and the line width, which leads to an increase in the linear range of the sensors. The sensitivity can be improved by increasing the number of strips appropriately, however, it is reduced when the line width increases. Smaller spacing results in a larger demagnetization factor due to the magnetic interactions between adjacent strips, which reduces the sensitivity of the sensor. The results obtained here from simulations and experiments are useful for designing magnetic sensors with similar structures.

## 1. Introduction

Miniature magnetic sensors are required for many emerging applications including consumer electronics, nondestructive testing, space exploration, and biomedical diagnostics [1,2,3,4,5,6]. Almost all the above applications require high sensitivity and high precision. Orthogonal fluxgate sensors with cores made of amorphous wire have small size and can be operated in fundamental mode by adding a proper DC bias to the AC excitation, which causes a dramatic reduction in Barkhausen noise [7,8,9,10]. One of the disadvantages of such wire-core orthogonal fluxgate sensors is the relatively low sensitivity caused by the small cross-sectional area of the wire. Li and Ripka et al. [11,12] studied a multi-wire-core orthogonal fluxgate sensor and found that the sensitivity of the sensor can be greatly increased. However, the linear measurement range of the multi-wire-core orthogonal fluxgate sensor was relatively small, reported in the literature [11] as about 10 μT, which limited its use in applications that require a wide measurement range such as magnetic diagnostics [13,14], large current contactless measurement [15,16], and so on.

The measurement range is subjected to the soft magnetic properties of the core and the demagnetizing field that depends on the core geometry. This means that adjustment of the geometry of the core to extend the measurement range must be considered carefully, since the soft magnetic properties of the core have to be maintained for sensitive measurement. A sensor with a shorter core has wider linear range as the effect of demagnetization is higher for the shorter core. However, shortening the core reduces the turns of the sensing coil, which leads to a rapid decrease in sensor sensitivity. It seems that it is hard to obtain both high sensitivity and wide measurement range.

In recent years, orthogonal fluxgate sensors based on amorphous ribbon cores have shown good performance [17,18,19]. In addition, compared with amorphous wire or multiwire cores, the ribbon core is more compatible with micro electro-mechanical system (MEMS) technology. Our previously reported MEMS meander-core orthogonal fluxgate sensor fabricated with Co-based amorphous ribbon exhibited high sensitivity and wide linear range [20]. The purpose of this paper is to study the demagnetization effect caused by the variations of the number of strips, the line width, and the spacing of the meander-cores, and further investigate the influence of the demagnetization factor on the sensitivity and linear range of the meander-core orthogonal fluxgate sensor in fundamental mode. We believe that this is the beginning of a systematic study of such sensors, which will provide hints for designers to manufacture sensors that meet application requirements.

## 2. Modeling and Simulation

### 2.1. Demagnetization Model

The importance of demagnetizing effect on the properties of a ferromagnetic object is well recognized. When the ferromagnetic object is exposed to a homogeneous longitudinal external magnetic field ***H***_0_, a demagnetizing field *D**M***, opposing the external field, is produced inside the ferromagnetic object. The magnetic field in the ferromagnetic object can be written as:

(1) $$H=H_{0}-DM$$

where *D* is a dimensionless demagnetization factor and ***M*** is the magnetization. In general, the demagnetization factor *D* is a tensor. However, the most interesting property for an orthogonal fluxgate sensor designer is the demagnetization factor in the direction of the sensor sensitivity axis. Thus, unless otherwise specified, only the demagnetization factor in the direction of the measured field will be discussed, which is a number between 0 and 1.

According to the work of Heimfarth [21], the output voltage of a fundamental mode orthogonal fluxgate sensor can be approximated as follows:

(2) $$V_{i}=-N_{2}\mu_{0}AH_{0}\frac{M_{s}\left(1-D\right)}{{\left(H_{e}\left(t\right)+M_{s}D\right)}^{2}}\frac{dH_{e}\left(t\right)}{dt}$$

where *N*_2_ is the number of sensing coil turns, *A* is the cross-sectional area of the sensor core, *µ*_0_ = 4π × 10^−7^ T·m/A is the permeability of vacuum, and ***M***_s_ is the core’s saturation magnetization. Circumferential excitation field ***H***_e_ (*t*) = ***H****_dc_* + ***H****_ac_* sin (2π*ft*) in the core is created by applied DC-biased AC excitation current.

Equation (2) clearly indicates that the voltage sensitivity of sensor can generally be increased by increasing the cross-sectional area of the core, but the limiting factor is the demagnetization factor *D*. The highest sensor output would be obtained for *D* = 0, and the sensor would yield an output of zero when *D* = 1. Moreover, it can be seen from Equation (1) that the total magnetic field acting on the core decreases due to the presence of a demagnetizing field; this corresponds to an increase in the linear measurement range of the sensor. Therefore, the linear measurement range of the sensor can be increased by increasing the demagnetization factor in the sensing direction.

An important fact is that the demagnetization factor is uniform only in the case of elliptical objects [22]. In all other cases, except ellipsoids, the demagnetization factor is a function of the position in the volume of the object. The demagnetization model solves the following equation for each point, r, iteratively coupled with Equation (1):

(3) $$H\left(r\right)\approx H_{0}-\overset{n}{\underset{i=1}{\sum }}D\left(r-r_{i}\right)\cdot M_{i}$$

Therefore, it is necessary to generalize the demagnetization factor to such objects with some sort of averaging. There are two common methods of averaging [23]: fluxmetric (or local) and magnetometric (or global) demagnetization factors. The fluxmetric demagnetization factor corresponds to an average of magnetization at the midplane of the object and is appropriate for measurement with a short sensing coil. The magnetometric demagnetization factor refers to an average of magnetization over the entire object and is appropriate for magnetometer measurement with a large sensing coil compared with the object’s dimensions. It indicates that the average demagnetization factor depends not only on the core geometry, but also on the geometry of the sensing coil. In our case, the sensing coil does not completely cover the core because the terminations of the core do not add significant signal. Therefore, when calculating the average demagnetization factor, only the magnetization of the core covered by the sensing coil is considered.

### 2.2. FEM Simulation

The average demagnetization factor can be calculated using the FEM (Finite Element Method) model of the demagnetizing field. FEM modeling can provide data on the magnetic field vector ***H*** and the magnetic flux density vector ***B*** at each point inside the magnetized ferromagnetic core. The data can be used to determine the demagnetization factor of any core shape. We will first use this model to calculate the demagnetization factor of a meander-core.

When considering the demagnetization factor in the measurement direction (*x*-axis in our case), only the *x*-axis components of the ***H***(*x*, *y*, *z*) and ***B***(*x*, *y*, *z*) vectors are used to calculate the demagnetization factor *D*. Supposing that the core is made of isotropic soft magnetic material. When a uniform constant external magnetic field ***H***_0_ is applied parallel to the core axis direction, the relation between the volume average of the core internal field strength *x*-axis component ***H****_x_* and the volume average of the core magnetization *x*-axis component ***M****_x_* is as follows:

(4) $$〈H_{x}〉=H_{0}-D〈M_{x}〉$$

The average magnetization ***M****_x_* inside the core can be expressed by:

(5) $$〈M_{x}〉=\frac{〈B_{x}〉}{\mu_{0}}-〈H_{x}〉$$

Combining Equations (4) and (5), the average demagnetization factor can be written as:

(6) $$D=\frac{〈H_{x}〉-H_{0}}{〈H_{x}〉-\left(〈B_{x}〉/\mu_{0}\right)}$$

**Figure 1:**
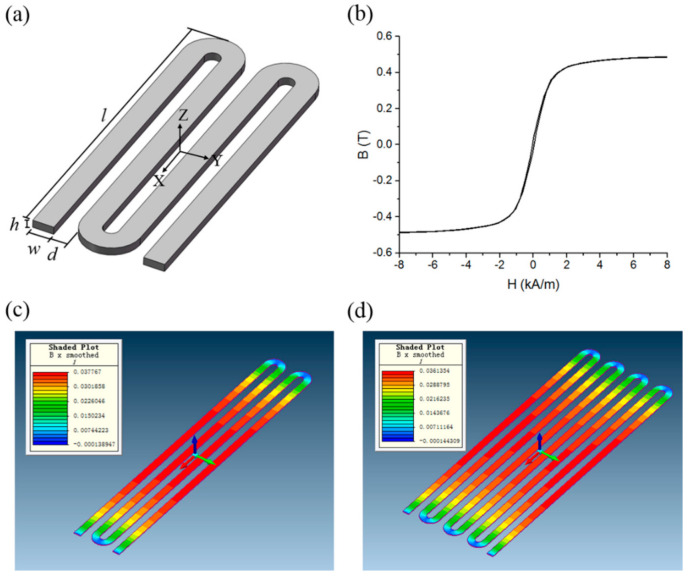
(**a**) Schematic view of the meander-core structure. (**b**) ***B***-***H*** curve of the Co-based amorphous ribbon used in the simulation. Magnet simulation results of magnetic flux density distribution of meander-core with: (**c**) four and (**d**) eight strips in x-direction (the length and line width of strips is 8 mm and 200 μm, respectively, the spacing between all the strips is 150 μm, and ***H***_0_ = 100 μT, applied in the x-axis).

**Figure 2:**
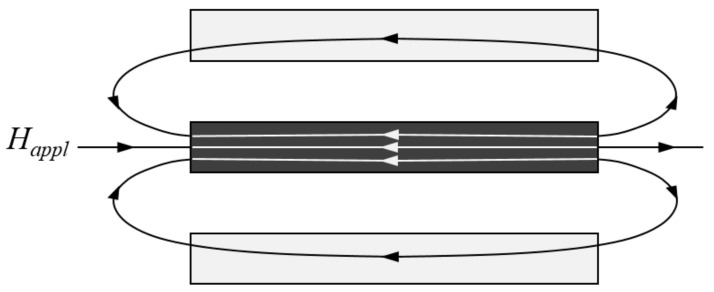
Schematic illustration of the magnetic field of a single strip in the meander-core. The strip is magnetized along the length direction. The magnetic field from the neighboring strips opposes the applied field inside the strip as seen by the white field lines, which will result in a decrease in the internal magnetic field.

Figure 1a shows a schematic view of the meander-core structure. Such a core is characterized geometrically by the length, *l*, line width, *w*, thickness, *h*, spacing between the strips, *d*, and the number of strips, *N*. The FEM simulation was implemented by MagNet software from Infolytica Corporation. The maximum mesh element size of magnetic core components and air regions are set at 20 μm and 500 μm, respectively, and the adaptive mesh element division function was used to generate the global model mesh. The ***B***-***H*** curve of the Co-based amorphous ribbon obtained from VSM (vibrating sample magnetometer) is shown in Figure 1b. The cores were exposed to an external homogeneous field ***B***_0_ = 100 μT (corresponding to ***H***_0_ ≈ 79.6 A/m). Two examples of the magnetic flux density distribution for the meander-core with four and eight strips in x-direction are shown in Figure 1c,d, respectively. It is easy to find that the magnetic flux density distribution of the meander core is not uniform. Along the longitudinal direction of the core, the flux density is large in the middle and small at both ends. Along the transverse direction of the core, the flux density of the middle strips is small and that of the edge strips is large. This is to be expected and simply explained by considering Figure 2. When the applied field is parallel with the longitudinal direction of meander-core, the stray field from the neighboring strips will tend to reduce the applied field due to the magnetization of each individual strip. Note that this entails the largest internal field being experienced by the outermost strips, whereas the minimum internal field is found at the center strips.

The nonuniform distribution of flux density in the core will lead to different demagnetization factors at each point of the core. The average demagnetization factor can be calculated from the results of the FEM analysis using Equation (6). Substituting the calculated demagnetization factor value into Equation (2) and combining other parameters (the saturation magnetization ***M****_s_* ≈ 400 kA/m, and the field strength in the core can be approximated by the ratio of ***H***/*I* = 2.5 kAm^−1^/A), the sensitivity of sensor can be achieved by computing the first harmonic amplitude of the resulting waveforms using Fourier analysis. The simulation results of average demagnetization factor and sensitivity are discussed in Section 4.

## 3. Experimental Details

Co-based amorphous ribbons (Co_66_Fe_4_Ni_1_B_14_Si_15_) were fabricated by quick quenching from melt onto a rotating barrel. The thickness of the ribbons was about 25 μm. The meander-core samples were prepared from the Co-based amorphous ribbons by photolithography and wet etching [24]. The fabrication processes were as follows: (a) fixing the polished amorphous ribbon on the surface of a clean glass substrate with epoxy glue; (b) spin-coating a 10 μm thick positive photoresist on the ribbon; (c) UV exposure for 20 s, rinsing in the developer for 90 s; (d) etching the ribbon sample in the acid mixture (HNO_3_:HCl:H_2_O_2_:H_2_O = 1:2:4:8) for about 5 min until the meander structure was clearly visible; (e) applying acetone solution to remove residual photoresist.

In the experiments, we prepared a series of meander-core samples with different geometries. Three sets of parameter variations were considered. Firstly, the number of strips in the meander-cores was varied (4, 8, 12, and 16) while the line width (200 μm) and spacing (150 μm) were kept constant. In addition, a single-strip core with a line width of 200 μm was designed for comparison. Secondly, the meander-core of eight strips was considered in which the line width was varied (100, 150, 200, 250, 300, 350, and 400 μm), and the spacing between the strips was fixed at 150 μm. Thirdly, the meander-core of eight strips with 200 μm line width was used, and the spacing between the strips was varied (50, 100, 150, 200, 250, 300, and 350 μm). The strip length of all the cores was 8 mm.

**Figure 3:**
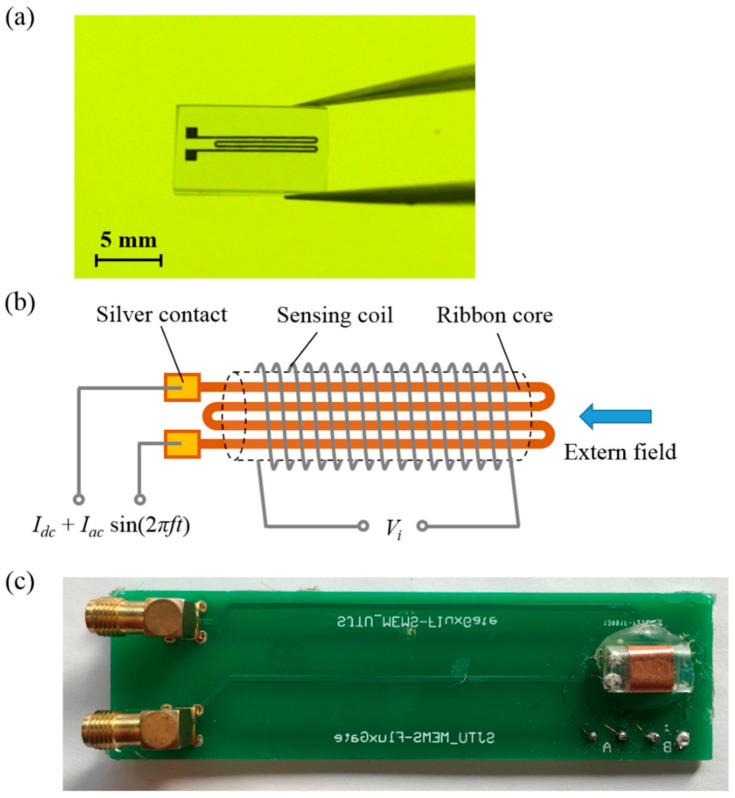
(**a**) Photograph of four-strip meander-core sample. (**b**) Schematic diagram of orthogonal fluxgate sensor head structure. (**c**) Photograph of orthogonal fluxgate sensor head with winding sensing coil.

Figure 3a shows a photograph of a four-strip meander-core sample with a line width of 200 μm and a spacing of 150 μm. The schematic diagram and photograph of the orthogonal fluxgate sensor head are shown in Figure 3b,c. The sensing coil with 100 turns of enameled copper wire, having a diameter of 60 μm, was wound on an acryl pipe of outer diameter 5 mm. The coil length was 7 mm, shorter than the core, and located in its center. Low-resistance pure silver was tightly bonded at each end of each structure as a conductive contact. The sensors were operated in the fundamental mode. The AC current was supplied by a Tektronix AFG 3022 function generator and amplified by a power amplifier (based on an OPA561). The DC bias was provided by a InsTek PST 3202 constant current source. The fundamental component of the output voltage was measured using the SR844 lock-in amplifier. The external magnetic field was provided by a Helmholtz coil connected to a high precision DC source, generating magnetic flux density ranging from 0 to 1 mT.

## 4. Results and Discussion

### 4.1. Varying the Number of Strips

**Figure 4:**
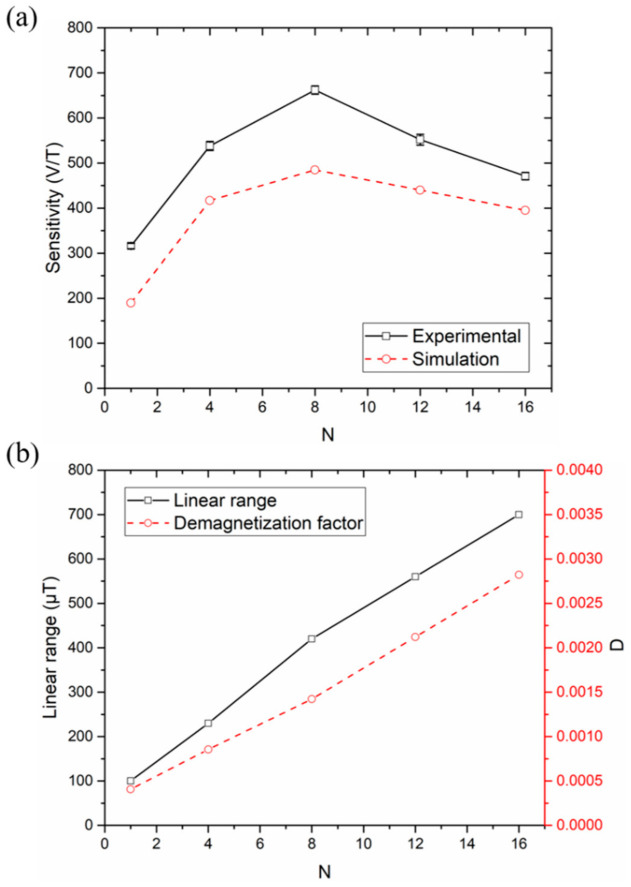
(**a**) The sensitivity of the experiment and simulation as a function of the number of strips. (**b**) The linear range as a function of the number of strips, and a comparison with the demagnetization factor *D*.

The prepared single-strip core and meander-cores with 4, 8, 12, and 16 strips were used as the core of the orthogonal fluxgate sensors. The DC bias was 200 mA, and the amplitude and frequency of AC current were selected as 150 mA and 200 kHz (see Figures S1 and S2 in Supplementary Materials). The sensitivity and linear measurement range was determined as the range in which the output response fit to a linear function with an R-squared value of 99%. Five independent measurements were repeated for each sensor. These settings would be used in the subsequent experiments. The solid lines in Figure 4a,b show the experimental sensitivity and linear range of the sensors as a function of the number of strips. The dotted lines in Figure 4a,b show the sensitivity and demagnetization factor calculated by simulation under the same excitation parameters for comparison.

According to the working principle of the orthogonal fluxgate sensor, the sensitivity of an orthogonal fluxgate sensor is positively correlated with the cross-sectional area of the magnetic core, and is inversely correlated with the demagnetization factor. Therefore, with an increase in the number of strips, the cross-sectional area of the sensing element increases proportionally, which improves the sensitivity of the sensor. However, the change in the number of strips will produce a demagnetization effect. From the simulation results of the demagnetization factor of the meander-cores in Figure 4b, it can be seen that the demagnetization factor increases with an increase in the number of strips. Therefore, we can expect that—with the same strip width and spacing—increasing the number of strips will result in an inflection point in the change trend of sensor sensitivity. The simulation and experimental test results in Figure 4a verified our expectations. As the number of strips increases, the sensitivity of the sensor first increases, and then decreases. 

For the core geometry we designed, when the number of strips is eight, the sensitivity measured in experiments reached the maximum value of 662 V/T. Compared with the sensitivity of the single-strip core sensor (316 V/T), the sensitivity of eight-strip meander-core sensor was 2.1 times higher. It is easy to understand that when the number of strips is less than eight, an increase in the cross-sectional area of the core has a greater influence on the sensitivity. When the number of the strips is greater than eight, the increase in demagnetization factor is the predominant effect. Of course, it should be pointed out that for different core geometries (such as length, line width, and spacing), the number of strips at the maximum sensitivity will be different. It mainly depends on which of the effects of the cross-sectional area and demagnetization factor are dominant. In addition, since the simulation sensitivity is an approximate value obtained by Equation (2), there is a deviation between the experimental and simulation results. However, the variation trend of the sensitivity is consistent, which proves the validity of the simulation results of the demagnetization factor.

It can be seen from Figure 4b that the linear range of the sensor increases almost linearly with the number of strips due to the increase in the demagnetization factor. As the number of strips increases from 1 to 16, the maximum linear range increases from 100 μT to almost 700 μT, which is about seven times higher. It can be expected that the linear measurement range of the sensor can be adjusted simply by changing the number of strips.

### 4.2. Varying the Line Width of Strips

**Figure 5:**
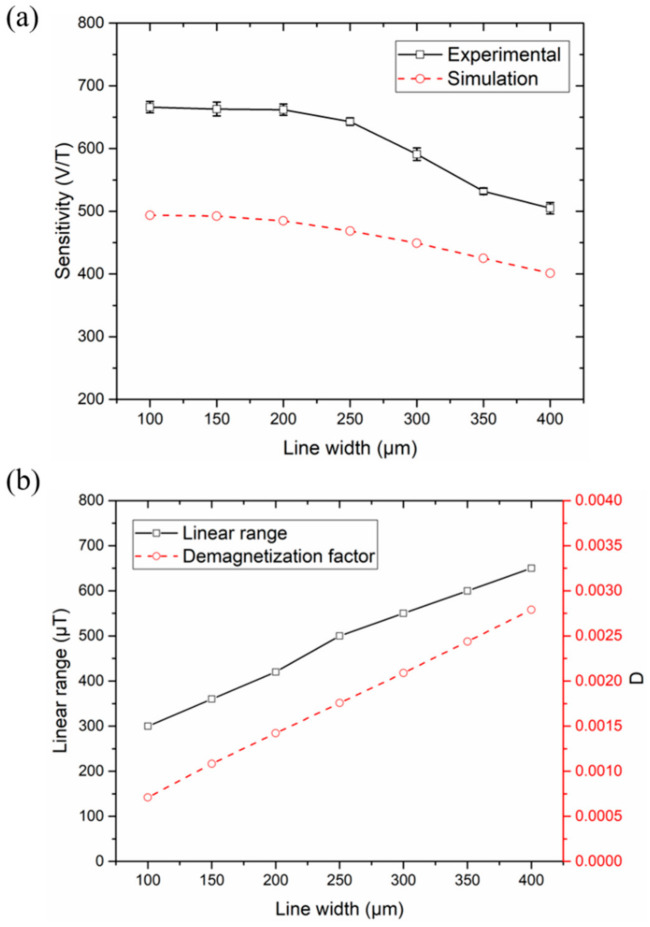
(**a**) The sensitivity of experiment and simulation as a function of the line width of strips. (**b**) The linear range as a function of the line width of strips, and comparison with the demagnetization factor *D*.

Eight-strip meander-cores with line width of 100, 150, 200, 250, 300, 350, and 400 μm were used to investigate the effect of line width variation on the sensitivity and linear range, and the results are shown in Figure 5a,b. The simulation results of sensitivity and demagnetization factor are also shown in the figure as a comparison. Similar to the change in the number of strips, an increase in the strip width will also increase the cross-sectional area of the sensing element, and it can be seen from the simulation result of Figure 5b that an increase in the strip width will lead to an increase in the demagnetization factor. Experimental results show that when the line width increased from 100 μm to 400 μm, the sensitivity of the sensor decreased from 666 V/T to 505 V/T, and the maximum linear measurement range increased from 300 μT to 650 μT. When the line width was less than 200 μm, the increase in cross-sectional area compensated for the effect of demagnetization, leaving the sensitivity almost constant. When the line width was greater than 200 μm, the demagnetization effect dominated, resulting in a rapid decrease in sensitivity.

The performance of the sensors was compared when the same cross-sectional area of the sensitive element was increased by increasing the number of strips and increasing the line width, respectively. Firstly, the line width was fixed at 200 μm, and the number of strips was increased from four to eight. As can be seen from Figure 4a,b, the sensitivity of the sensors increased by 23% (from 538 V/T to 662 V/T), and the maximum linear range increased by 83% (from 230 μT to 420 μT). Then, the number of strips was fixed at eight and the line width was increased from 100 μm to 200 μm. It can be seen from Figure 5a,b that the sensitivity of the sensor was almost unchanged, and the maximum linear range increasesd by 40% (from 300 μT to 420 μT). Obviously, when the increased cross-sectional area is the same, the changes in sensitivity and linear range caused by increasing the line width is far less than the change caused by increasing the number of strips.

### 4.3. Varying the Spacing between Strips

**Figure 6:**
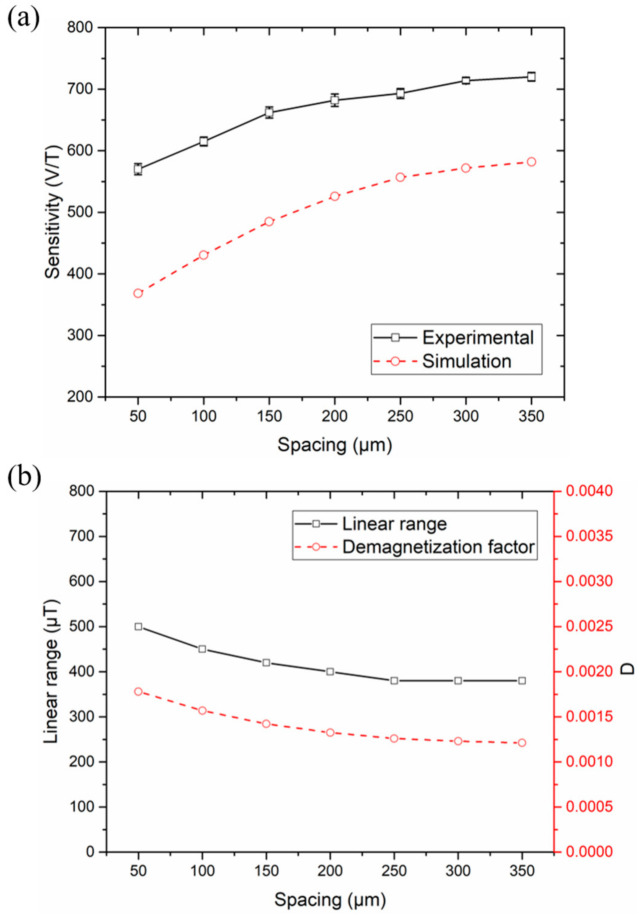
(**a**) The sensitivity of experiment and simulation as a function of the spacing between strips. (**b**) The linear range as a function of the spacing between strips, and the comparison with the demagnetization factor *D*.

Figure 6a,b provide a clear view of the variation of sensitivity and linear range, with the spacing between strips increasing from 50 μm to 350 μm for the eight-strip meander-core sensors. As well as the data for the different spacing, the simulation results of sensitivity and demagnetization factor are also presented. Different from the results caused by changes in the number of strips and line width, the demagnetization factor first decreased rapidly and then tended to be flat with the increase in spacing. The change in linear range is basically consistent with the demagnetization factor, whereas the change in sensitivity is opposite. When the strip spacing was as small as 150 μm, the demagnetization factor changed more significantly. This phenomenon also verifies the analysis results in Figure 2. When the strip spacing was small, the influence between adjacent strips increased, the internal magnetic field of the strips became smaller, and the demagnetization effect became more intense. When the spacing became larger, the demagnetization effect weakened. Therefore, when designing meander-cores, the spacing should not be made too small to achieve sufficient sensor performance.

In summary, all the mechanisms (the number of strips, line width, and spacing) that influence the demagnetization factor will result in changes in the performance of the orthogonal fluxgate sensor. In addition, the length, thickness, and surface roughness of the ribbon core will also affect the demagnetization factor. We must select structural parameters reasonably, and ensure that the sensor’s comprehensive performance is optimal while ensuring the miniaturization of the sensor.

**Figure 7:**
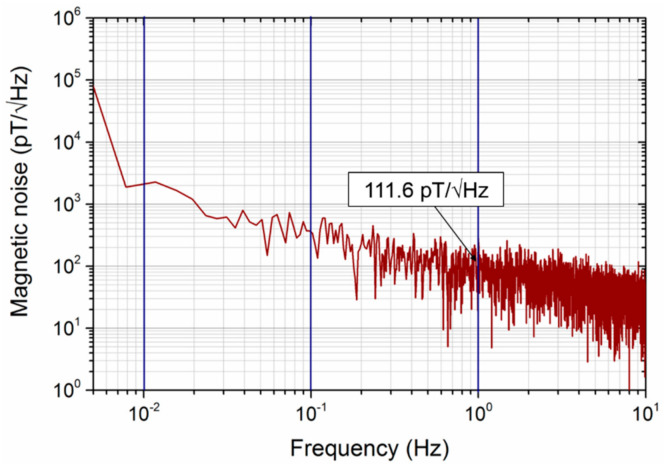
The magnetic noise spectrum of an eight-strip meander-core orthogonal fluxgate sensor working in fundamental mode.

In addition to the sensitivity and linear range, the signal-to-noise ratio is also a key factor in evaluating the performance of sensors in practical applications. A meander-core orthogonal fluxgate sensor with line width of 200 μm, spacing of 150 μm, and strip number of 8 was selected, and its noise level in the fundamental mode was tested in a shielded environment. The sensor head was placed inside a magnetic shielding cylinder, which consisted of six layers of soft magnetic ribbons, and the axis direction of the shield was perpendicular to the earth magnetic field. Figure 7 shows the equivalent magnetic noise spectrum of the sensor under 150 mA AC excitation at 200 kHz and 200 mA DC bias. The measured equivalent magnetic noise was about 111.6 pT/√Hz at 1 Hz, and the rms noise was 582.7 pT in the frequency range of 0.1–10 Hz. Some recently reported orthogonal fluxgate sensors focus on reducing noise [25,26]. Janosek et al. [27] reported that the noise level of an orthogonal fluxgate based on amorphous wire core reached 1 pT/√Hz at 1 Hz, but the maximum linear range was 12 μT, and the length of the core was more than 800 mm. The meander-core orthogonal fluxgate sensor shows good comprehensive performance while maintaining a small size. Moreover, by adjusting the structural parameters of the meander-core, the sensor can obtain a wide adjustable linear measurement range, which has a wide application prospect in some occasions where linear range is required.

## 5. Conclusions

The average demagnetization factor of the orthogonal fluxgate sensor with meander-core was modelled by FEM. Based on the demagnetization factor value obtained from the model simulation, the sensitivity of the meander-core orthogonal fluxgate in the fundamental was evaluated. The sensitivity of the measurements on the meander-core orthogonal fluxgate sensors by varying structure parameters (the number of strips, line width, and spacing) are in agreement with the simulation sensitivity results. The change trend of linear range were consistent with the demagnetization factor. This model provides a convenient solution for quickly estimating the demagnetization factor and sensitivity of a given orthogonal fluxgate sensor with meander-core, prior to manufacturing and measuring of the actual core.

## Data Availability

The data presented in this study are available in the paper and Supplementary Materials.

## Supplementary Materials

The following are available online at https://www.mdpi.com/article/10.3390/mi12080937/s1, Figure S1: the relationship between sensor sensitivity and the DC and AC components of the excitation current, Figure S2: dependence of the sensitivity of the sensor on the excitation frequency for the single strip core and meander-cores with four, eight, 12, and 16 strips.
