# Supplementary material for: Demagnetization Effect in a Meander-Core Orthogonal Fluxgate Sensor
Source: Micromachines (Basel). 2021 Aug 9;12(8):937. doi: 10.3390/mi12080937 (PMC8400970; doi:10.3390/mi12080937)
Supplement: Supplementary file 1 [file micromachines-12-00937-s001.zip › micromachines-1324088-supplementary.pdf]

micromachines

Supplementary Materials

Demagnetization Effect in a Meander-Core Orthogonal Fluxgate Sensor

Shaotao Zhi [1], Xuecheng Sun [2,*], Qiaozhen Zhang [1], Jie Chen [1], Xiangfen Zhang [1], Hongyu Chen [2] and Chong Lei [3,*]

1 College of Information, Mechanical and Electrical Engineering, Shanghai Normal University, Shanghai 200234, China; zhist@shnu.edu.cn (S.Z.); zhangqz@shnu.edu.cn (Q.Z.); jiechen@shnu.edu.cn (J.C.); xiangfen@shnu.edu.cn (X.Z.)
2 Research and Development Center of Microelectronics, School of Mechatronic Engineering and Automation, Shanghai University, Shanghai 200444, China; mark_white@shu.edu.cn
3 Key Laboratory of Thin Film and Microfabrication Technology (Ministry of Education), Department of Micro/Nano Electronics, School of Electronic Information and Electrical Engineering, Shanghai Jiao Tong University, Shanghai 200240, China
* Correspondence: sunxc@shu.edu.cn (X.S.); leiqhd@sjtu.edu.cn (C.L.)

Citation: Zhi, S.; Sun, X.; Zhang, Q.; Chen, J.; Zhang, X.; Chen, H.; Lei, C. Demagnetization Effect in a Meander-Core Orthogonal Fluxgate Sensor. *Micromachines* **2021**, *12*, x. https://doi.org/10.3390/mi12080937

Academic Editors: Jayne C Garno

Received: 17 July 2021
Accepted: 6 August 2021
Published: 9 August 2021

Publisher's Note: MDPI stays neutral with regard to jurisdictional claims in published maps and institutional affiliations.

Copyright: © 2021 by the authors. Submitted for possible open access publication under the terms and conditions of the Creative Commons Attribution (CC BY) license (http://creativecommons.org/licenses/by/4.0/).

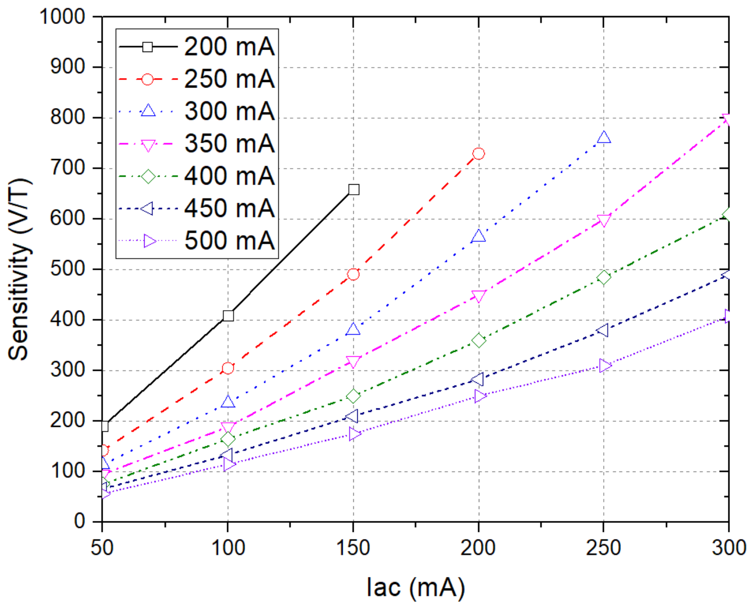

Figure S1. The relationship between sensor sensitivity and the DC and AC components of the excitation current.

The excitation current of the fundamental mode orthogonal fluxgate sensor contains DC and AC component, so the relationship between excitation current and sensitivity is complex. Taking the orthogonal fluxgate sensor with eight-strip meander-core as an example, we tested the relationship between its sensitivity and the DC and AC components of the excitation current, as shown in Figure S1. The abscissa represents the AC component and each curve represents the DC component, and the frequency of AC current was 200 kHz. The results show that the sensitivity increases with the increase of AC and decreases with the increase of DC. When DC and AC increase at the same time, the sensitivity is higher. However, the excitation current cannot be increased indefinitely, which will cause the magnetic core to overheat and produce additional noise. Therefore, the optimal excitation parameters are usually determined by the relationship between noise and excitation parameters. We will continue to study it in our future work. In this paper, in order to more directly compare the influence of demagnetization on the simulation and experimental results of the sensitivity, we set the excitation parameters of sensors with different

magnetic cores to the same, and the amplitudes of DC and AC components are 200 mA and 150 mA respectively.

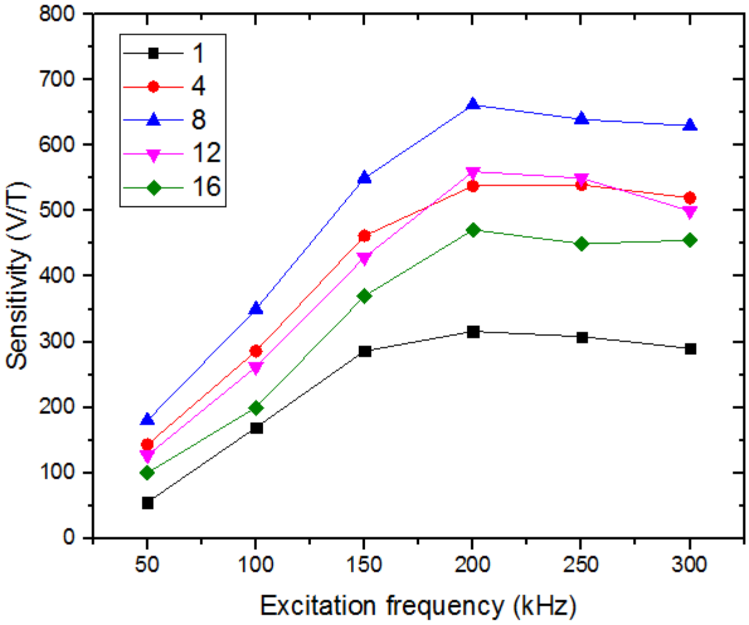

Figure S2. Dependence of the sensitivity of the sensor on the excitation frequency for the single strip core and meander-cores with four, eight, 12, and 16 strips.

In order to study the response frequency of orthogonal fluxgate sensor, we used the single strip and meander-shaped cores with 4, 8, 12 and 16 strips as the core of the orthogonal fluxgate sensors. The amplitude of AC current and DC bias were 150 mA and 200 mA, respectively. And the frequency of AC current varied from 50 kHz to 300 kHz. Figure S2 shows the excitation frequency dependences of the sensor sensitivity for the cores with one, four, eight, 12 and 16 strips. The results show that the resonant frequency is independent of the number of strips in the cores, and it is always around 200 kHz for all the sensors. This is because the resonant frequency depends on the parasitic capacitance and inductance of the sensing coil, and the sensing coils in all the sensors are the same. In addition, the excitation frequency of 200 kHz is a relatively low frequency, and the difference in the inductance caused by the change of the number of strips is small.
